# Lasing microbottles

**DOI:** 10.1038/lsa.2017.102

**Published:** 2017-10-06

**Authors:** Misha Sumetsky

**Affiliations:** 1 Aston Institute of Photonic Technologies, Aston University, Birmingham B4 7ET, UK

**Lasing of an optical microbottle resonator at predetermined resonant wavelengths is feasible via spatial engineering of the pump laser beam.**

The whispering gallery mode (WGM) phenomenon, which was first discovered by Lord Rayleigh in 1912 for acoustic waves running around the interior of the dome of St Paul’s Cathedral in London^1^, has found exciting microscale applications for optical microresonators. Similar to acoustic WGMs, optical WGMs circulate inside an optical microresonator, reflecting from its surface due to the effect of total internal reflection. Different types of optical WGM microresonators enable the delay, control, generation and transformation of light on a microscale; these types have been demonstrated and investigated^2, 3, 4^. Their fabrication methods include the melting of optical materials, mechanical and laser machining and polishing, and the use of liquid or solidified liquid droplets. These methods allow for the fabrication of microresonators possessing the highest Q-factor (that is, the smallest attenuation of light) required for applications.

One of the important applications of WGM microresonators is the fabrication of miniature lasers. The first demonstration of a WGM spherical laser with an ~1-mm radius was published more than 50 years ago^5^ and was followed by demonstrations of much smaller microlasers fabricated from microsphere, microtoroid and microbottle resonators^2, 3, 4^. Usually, the bandwidth of the fluorescence spectrum generated by the pump light is wider than the microresonator’s free spectral range, resulting in the observation of the multi-resonance lasing. To reduce the number of lasing modes and, in particular, to achieve the single mode lasing, adjusting the position of the input pump light delivered to the microresonator through a transverse microfibre taper or focused laser beam has been suggested^6, 7^. This approach allows a reduction in the number of lasing WGMs but lacks universality.

Recently, Gu *et al.*^8^ writing in *Light: Science & Applications*, have made a significant step forward in both the fabrication of microlasers and the application of microbottle resonators by suggesting and demonstrating the spatial engineering of the input pump light. Instead of adjustment of the coordinates of the localized pump input, which was explored previously^6, 7^, they proposed to generate lasing of the selected WGMs by optimizing the distribution of the pump light along the microresonator axis. To demonstrate this idea, the authors explored a microbottle resonator that, as they suggested, and, as is discussed below, is most suitable for this application and is essential for the future research and development of microlasers.

To better understand the ideas of Gu *et al.* it is instructive to compare the microsphere, microtoroid and microbottle resonators. Microsphere resonators have a successful multidecade history of research, development and applications^2, 3, 4^ starting with the pioneering work^5^. Similar to microsphere resonators, the microtoroid resonators introduced in works^9, 10^ have found many exciting applications, ranging from optical signal processing, lasing and frequency comb generation to quantum networking and ultra-precise sensing^2, 3, 4^. Microbottle resonators introduced more than a decade ago^11^ have promising applications as nonlinear switchers^12^, microlasers^7, 13^, miniature delay lines^14^, frequency comb generators^15^ and quantum processors^16^. The principal difference among microsphere, microtoroid and microbottle resonators is illustrated in Figure 1. The axially symmetric versions of these devices are characterized by an axial radius, *r*_ax_, and an azimuthal radius, *r*_az_. For resonators with nearly spherical shapes (Figure 1a), *r*_ax_ and *r*_az_ are comparable, *r*_ax_~*r*_az_. For microtoroid resonators (Figure 1b), the axial radius is relatively small, *r*_ax_<<*r*_az_. Alternatively, for microbottle resonators (Figure 1c), the axial radius is typically much greater than the azimuthal radius, *r*_ax_>>*r*_az_, and can be very large, occasionally exceeding a kilometer^14^. Consequently, as illustrated in Figure 1, the distribution of WGMs along the axis of a microbottle resonator can have a characteristic variation length greatly exceeding those of microsphere and microtoroid resonators. This result significantly simplifies the external access to these WGMs and makes the microbottle resonator very attractive for various applications.

In the experiment^8^, the pump field spatial distribution was determined by the interference pattern of an input laser beam at wavelength *λ*=532 nm. The period of the pattern varied from 0.7 to 3 μm. The concept of spatial pump engineering suggests that the distribution of the input field can be appropriately adjusted to maximize lasing at predetermined resonance wavelengths. To this end, it is essential for the characteristic spatial variation length of the input field, Δ*z*_in_, to be smaller (ideally, much smaller) than that of the WGMs in the microresonator. While the smallest possible Δ*z*_in_ is determined by the diffraction limit, Δ*z*_in_>*λ*/2, the characteristic variation length of WGMs along the microresonator axis can be estimated as follows. Assume that the variation of the microresonator radius along axis *z* is parabolic, *ρ*(*z*)=*r*_az_−*z*^2^/(2*r*_ax_). Then, the distribution of the WGM amplitude along *z* is found to be 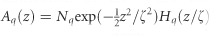. Here, *H*_*q*_(*x*) is the Hermite polynomial, *N*_*q*_ is the normalization factor, *ζ*=8^−1/4^(π*n*)^−1/2^*λ*^1/2^(*r*_az_*r*_ax_)^1/4^, and *n* is the refractive index of the microresonator material. From this expression, the characteristic variation length of *A*_*q*_(*z*) is found to be Λ_*q*_=(2*q*+1)^−/2^*ζ*. Figure 1d shows the axial distribution of the power *P*_*q*_(*z*)=*A*_*q*_(*z*)^2^ of the WGMs for the quantum numbers *q*=0,1,2,3. For comparison, this distribution is rescaled for the microsphere, microtoroid and microbottle resonators in Figure 1a–1c. Consider a microsphere resonator with *r*_ax_~*r*_az_~50 μm and *n*~1.5. At a radiation wavelength of *λ*~0.5 μm the WGMs of this resonator have Λ_0_~1.4 μm and Λ_3_~0.52 μm. For the microbottle resonator investigated by Gu *et al.*, *r*_ax_~22.5 μm and *r*_az_~2.4 μm such that Λ_0_~0.53 μm and Λ_3_~0.2 μm. Though these values of Λ_*q*_ are smaller than those in the example of a microsphere resonator, the spectrum of the microbottle resonator is much simpler (due to the small *r*_az_~2.4 μm) and, therefore, easier to handle. Crucially, the fabrication of microbottle resonators with much larger axial radii and, thus, much larger Λ_*q*_ is possible. For example, for the microbottle resonator with an exceptionally large *r*_ax_~1.6 km and a relatively small *r*_az_~19 μm^14^, the characteristic axial length of the WGMs is two orders of magnitude greater: Λ_0_~81 μm and Λ_3_~31 μm. For this resonator, the condition Δ*z*_in_<<Λ_*q*_ is satisfied for all practically achievable values of *q*, and thus, the spatial pump engineering is much easier.

Looking forward, the idea of spatial pump engineering of Gu *et al.* promises to attract appreciable attention from researchers working on the development and application of optical microresonators and, in particular, microlasers. Several interesting questions remain unanswered. In particular, the theory of spatial pump engineering, which has not yet been developed, has to answer the question of how to design the profile of an input laser beam so that it initiates lasing of a predetermined single WGM or several WGMs. Remarkably, the idea of spatial engineering can be directly applied to the excitation of predetermined WGMs in a passive microbottle resonator. In fact, to excite a single WGM, the distribution of the amplitude of the input beam along the resonator axis should be proportional to the amplitude *A*_*q*_(*z*) of this WGM. Then, other WGMs will not be excited because functions *A*_*q*_(*z*) with different *q* are orthogonal. Finally, Figure 1e presents a prospective device inspired by the work of Gu *et al.* The premise of the device is based on bridging the method of spatial pump engineering with the Surface Nanoscale Axial Photonics (SNAP) platform. The SNAP platform enables the fabrication of microbottle resonators with a predetermined spectrum and nanoscale variations of their azimuthal radius *ρ*(*z*) with unprecedented subangstrom precision^14, 17^. The unwanted WGMs can be attenuated in the fabricated microresonator using the spectral cleaning method^18^. To minimize the power consumption, the input beam can be delivered into the microbottle resonator evanescently through a prism. The evanescent coupling of light assumes that the gap between the prism and the microbottle is on the order of 100 nm and is kept constant along the microbottle axis. This condition does not prevent variations in the microbottle radius along its axis on the nanometer scale, as implemented in SNAP. Thus, it is hypothesized that the device illustrated in Figure 1e, which makes use of the original premise of Gu *et al.* is feasible.

## Figures and Tables

**Figure 1:**
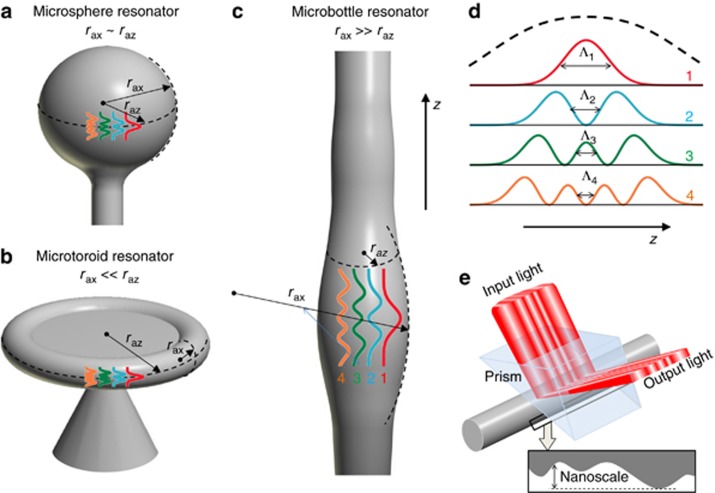
Illustration of microsphere (**a**), microtoroid (**b**) and microbottle (**c**) resonators. (**d**) The characteristic axial distributions of the WGM power in these resonators for axial quantum numbers *q*=0(red), *q*=1 (blue), *q*=2 (green) and *q*=3 (yellow) are shown, as well as those for microsphere, microtoroid and microbottle resonators in (**a**–**c**). (**e**) Illustration of a microresonator device based on the bridging of the spatial engineering of the input light and the SNAP platform.
